# Targeting Zfp148 activates p53 and reduces tumor initiation in the gut

**DOI:** 10.18632/oncotarget.10899

**Published:** 2016-07-28

**Authors:** Anna Nilton, Volkan I. Sayin, Zhiyuan V. Zou, Sama I. Sayin, Cecilia Bondjers, Nadia Gul, Pia Agren, Per Fogelstrand, Ola Nilsson, Martin O. Bergo, Per Lindahl

**Affiliations:** ^1^ Wallenberg Laboratory, Department of Molecular and Clinical Medicine, Institute of Medicine, Gothenburg, Sweden; ^2^ Department of Biochemistry, Institute of Biomedicine, Gothenburg, Sweden; ^3^ Sahlgrenska Cancer Center, Institute of Biomedicine, Department of Pathology and Genetics, Gothenburg, Sweden; ^4^ Sahlgrenska Cancer Center, Department of Molecular and Clinical Medicine, Institute of Medicine, Sahlgrenska Academy at the University of Gothenburg, Gothenburg, Sweden

**Keywords:** intestinal tumors, tumor suppressor p53, apoptosis

## Abstract

The transcription factor Zinc finger protein 148 (*Zfp148, ZBP-89, BFCOL, BERF1, ht*β) interacts physically with the tumor suppressor p53, but the significance of this interaction is not known. We recently showed that knockout of *Zfp148* in mice leads to ectopic activation of p53 in some tissues and cultured fibroblasts, suggesting that Zfp148 represses p53 activity. Here we hypothesize that targeting Zfp148 would unleash p53 activity and protect against cancer development, and test this idea in the *APC^Min/+^* mouse model of intestinal adenomas. Loss of one copy of *Zfp148* markedly reduced tumor numbers and tumor-associated intestinal bleedings, and improved survival. Furthermore, after activation of β-catenin-the initiating event in colorectal cancer-*Zfp148* deficiency activated p53 and induced apoptosis in intestinal explants of *APC^Min/+^* mice. The anti-tumor effect of targeting Zfp148 depended on p53, as *Zfp148* deficiency did not affect tumor numbers in *APC^Min/+^* mice lacking one or both copies of *Trp53*. The results suggest that Zfp148 controls the fate of newly transformed intestinal tumor cells by repressing p53 and that targeting Zfp148 might be useful in the treatment of colorectal cancer.

## INTRODUCTION

The transcription factor Zinc finger protein 148 (*Zfp148, ZBP-89, BFCOL, BERF1, ht*β) interacts physically with the tumor suppressor p53, but the significance of this interaction is not known [1]. We have recently shown that *Zfp148* deficiency leads to ectopic activation of p53 in mice and cultured fibroblasts, suggesting that Zfp148 represses p53 activity [2]. Knockout of *Zfp148* leads to respiratory distress and partial neonatal lethality in mice that are caused by proliferative arrest of pulmonary cells, and to premature senescence in cultured mouse embryonic fibroblasts. The phenotypes are rescued by deletion of one or two copies of *Trp53* (the gene encoding mouse p53). Moreover, loss of one copy of *Zfp148* reduces proliferation of tissue macrophages and atherosclerosis in *Apoe^−/−^* mice by increasing p53 activity [3]. Otherwise mice lacking one copy of *Zfp148* are fertile and healthy [2, 4].

Several studies show that Zfp148 is required for the integrity of intestinal epithelium suggesting a possible link to colorectal cancer (CRC) [5-8]. Moreover, Zfp148 expression is increased in human CRC compared to normal mucosa [9]. CRC is one of the most common cancer forms and a leading cause of cancer death in the Western world [10]. 80 percent of CRCs are caused by inherited or somatic mutations in the adenomatous polyposis coli (*APC*) gene [11]. *APC* mutations give rise to adenomatous polyps that progress to carcinomas, a process driven by additional mutations including *p53*-mutations [12]. The *APC^Min/+^* mouse harbours a mis-sense mutation in the *APC* locus which leads to the production of a truncated APC protein [13]. The mutated APC protein lacks the β-catenin binding domain, which leads to an accumulation of β-catenin in the nucleus and activation of β-catenin target genes [14]. *APC^Min/+^* mice develop numerous adenomatous polyps, predominantly in the small intestine, which leads to intestinal bleedings, anemia and death at about 30 weeks of age [13]. The model is therefore appropriate for studies of early-stage CRC.

Our finding that *Zfp148* deficiency activates p53 raises the possibility that Zfp148 promotes cancer by repressing p53 activity. In line with this, high expression of Zfp148 in oesophageal squamous cell cancer and clear cell renal cell carcinoma correlates with cancer progression and poor prognosis [15, 16]. However, the role of Zfp148 and its impact on p53 activity in cancer remains largely unknown. Here we address the role of Zfp148 in the *APC^Min/+^* model of CRC, and hypothesize that reduced expression of Zfp148 confers protection against intestinal adenomas by unleashing p53 activity. For this, we crossed *Apc^Min/+^* mice with mice lacking Zfp148 (*Zfp148^gt/gt^* and *Zfp148^gt/+^*) and studied the development of intestinal adenomas.

## RESULTS

**Figure 1:**
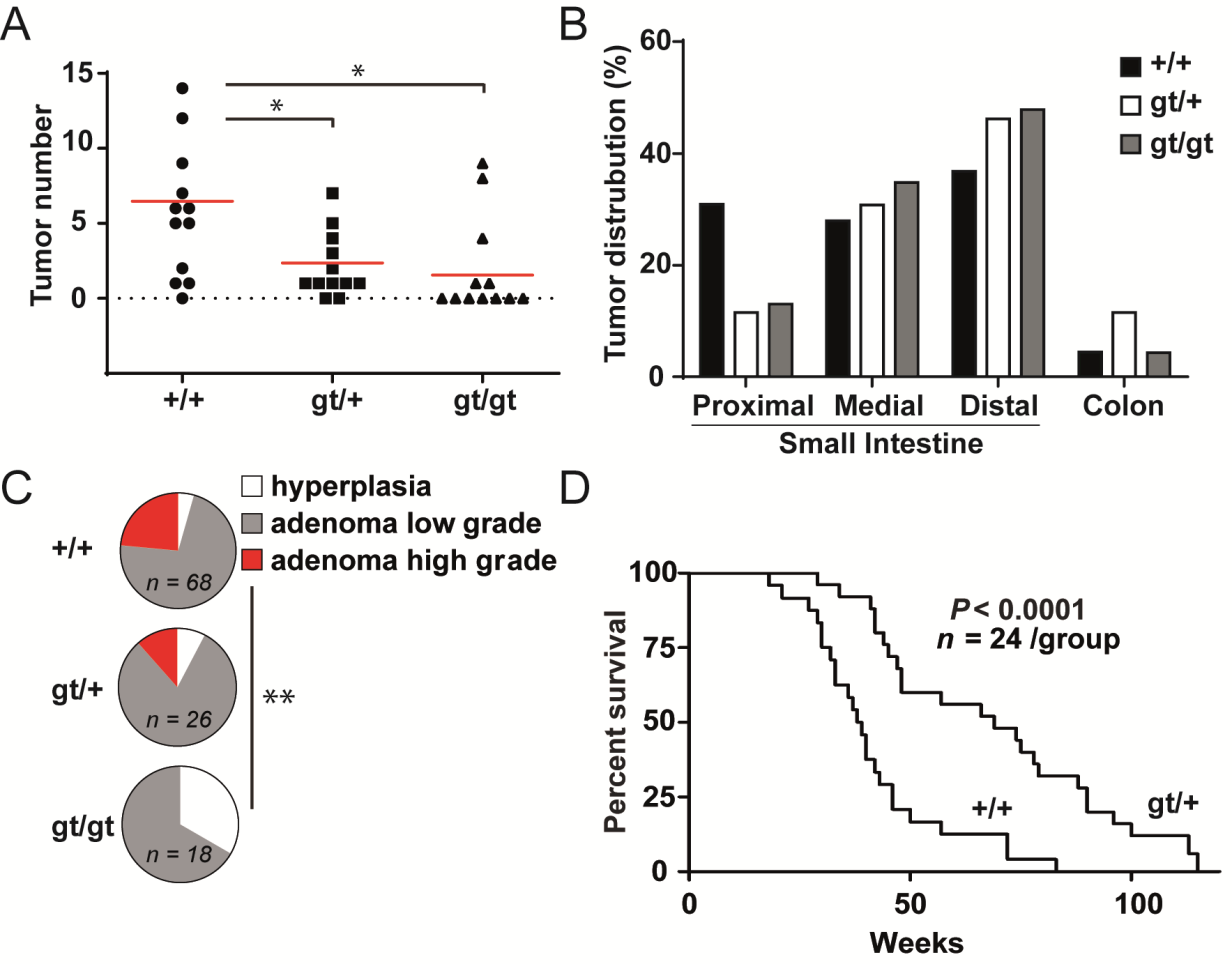
*Zfp148* deficiency reduces tumor growth in mice with *Apc*-induced intestinal adenomas **A.** Tumor count in intestines from *Apc, ApcZfp148*, and *ApcZfp148* mice at 12 weeks of age (*n* = 12). **B.** Percentage of tumors located in proximal, medial and distal third of small intestines and colon of *Apc, ApcZfp148*, *ApcZfp148* mice at 12 weeks of age. **C.** Tumor stage (hyperplasia, low grade adenoma, high grade adenoma) in intestines from *Apc, ApcZfp148*, *ApcZfp148* mice at 12 weeks of age (*n* = 23-68 tumors in intestines from 12 mice per group). **D.** Kaplan-Meier plot showing overall survival of *Apc* and *ApcZfp148* mice (*n* = 24 per group). Data are represented as mean ± SEM. *P < 0.05, **P < 0.01.

We established *Zfp148^gt/+^* and *Zfp148^gt/gt^* mice on the *APC^Min/+^* genetic background (from here on designated *Zfp148^gt/+^* and *Zfp148^gt/gt^* mice, respectively) to investigate if Zfp148 is involved in intestinal adenoma formation. Intestines from 12 week old mice were sectioned and stained with haematoxylin and eosin for evaluation of tumor development. Deletion of one or two copies of *Zfp148* markedly reduced tumor numbers compared to *APC^Min/+^* controls, without significantly altering the anatomical distribution of tumors (Figure 1A, 1B). Moreover, the tumors in *Zfp148^gt/gt^* mice were of lower histopathogical grade compared to *APC^Min/+^* controls (Figure 1C). Thus, Zfp148 is required for the formation of intestinal adenomas.

*APC^Min/+^* mice primarily die from secondary phenotypes including severe, chronic anaemia [17]. Since levels of haemoglobin and hematocrit were higher in *Zfp148^gt/+^* and *Zfp148^gt/gt^* mice compared to controls, and the frequency of intestinal bleedings was lower (Supplementary Figure 1A-1C), we argued that deletion of *Zfp148* may extend the survival of these mice. Indeed, loss of a single copy of *Zfp148* extended the median survival time of *Apc^Min/+^* mice with 69%, from 39 to 66 weeks (Figure 1D). Because the majority of *Zfp148^gt/gt^* mice die before 6 months of age, most from respiratory distress and some for unknown reasons [2], we did not set up *Zfp148^gt/gt^* mice in the survival study or in mechanistic studies since the results could be skewed by confounding phenotypes.

**Figure 2:**
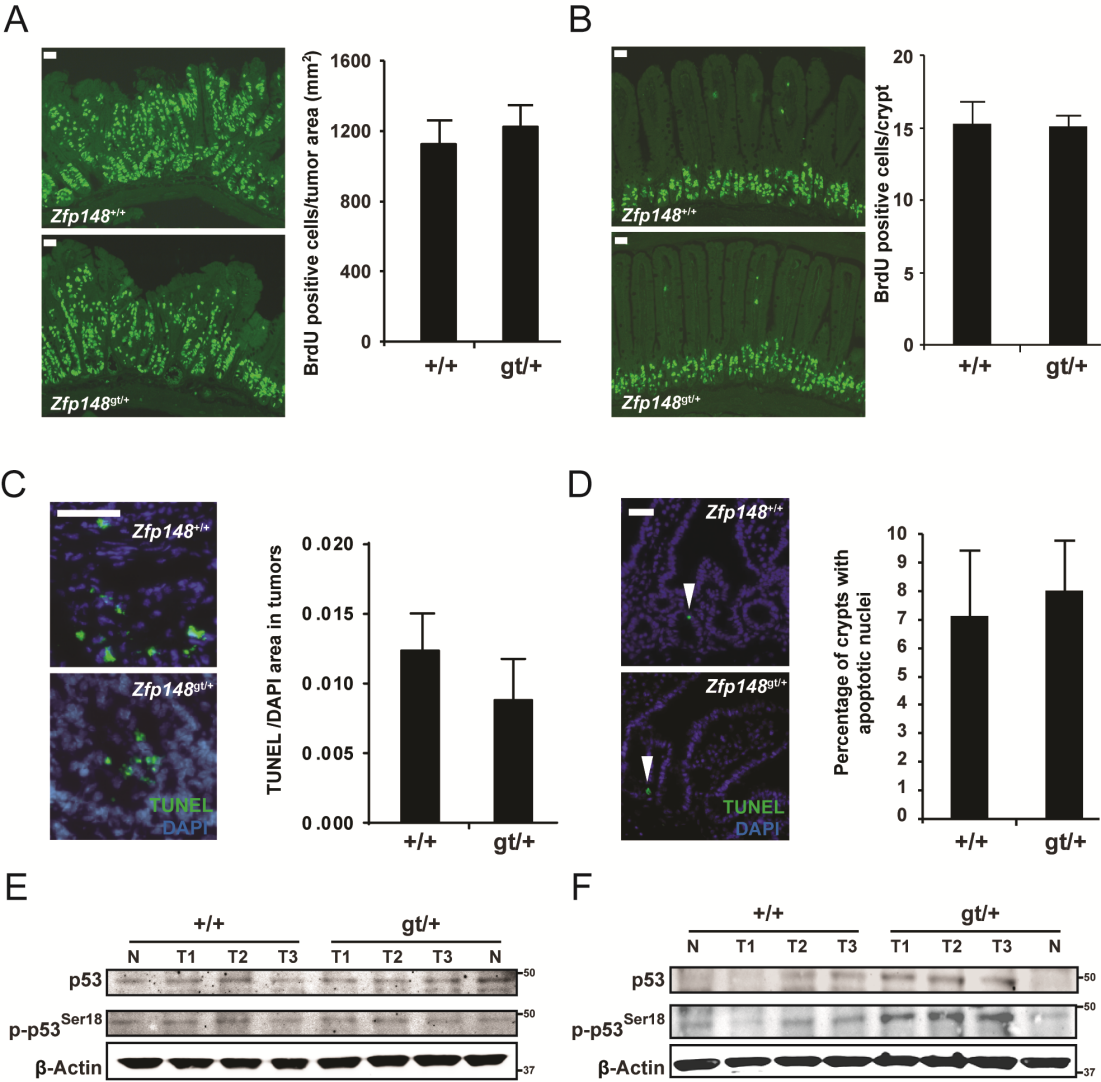
Tumor growth rate is not affected in *ApcZfp148* mice **A.**-**B.** Left: Representative immunofluorescence micrographs showing BrdU-positive cells in tumor tissue **A.** and normal intestinal epithelium **B.** in *Apc*and *ApcZfp148* mice. BrdU was injected into the peritoneal cavity 2 hours before the mice were sacrificed. Right: Quantification of BrdU-positive cells in tumor tissue (A; BrdU-positive cells per tumor area; *n* = 11-18 tumors in intestines from 5-8 mice per group) and normal intestinal epithelium (B; average number of BrdU-positive cells per crypt; *n* = 60 crypts per mouse in 5-7 mice per group). **C.**-**D.** Left: Immunofluorescence micrograph showing TUNEL-positive cells (green) in tumor tissue **C.** and normal intestinal epithelium **D.** in *Apc*and *ApcZfp148* mice. Nuclei are stained blue with DAPI (4′,6-diamidino-2-phenylindole). Right: Quantification of TUNEL-positive cells in tumor tissue (C; TUNEL-positive area per DAPI-positive area; *n* = 8-9 tumors per group) and normal intestinal epithelium (D; percentage of crypts with TUNEL-positive cells; *n* = 6-7 mice per group). **E.**-**F.** Western blots of phosphorylated and total p53 in tumors dissected from 12 weeks old **E.** and 24 - 26 weeks old **F.** *ApcZfp148* and *Apc*mice (*n* = 3). β-Actin was used as loading control. Data are represented as mean ± SEM. *P < 0.05, **P < 0.01. Scale bars, 50μm.

The extended survival and lower tumor burden of *Zfp148*-deficient mice raise the possibility that *Zfp148* deficiency reduces tumor growth rate. However, as demonstrated by bromodeoxyuridine (BrdU) incorporation, expression of proliferating nuclear cell antigen (PCNA), and terminal deoxynucleotidyl transferase dUTP nick end labeling (TUNEL), there was no difference in cell proliferation or apoptosis of tumor cells or normal crypt epithelial cells between 12 weeks old *Zfp148^gt/+^* mice and controls, respectively (Figure 2A-2D and Supplementary Figure 2A, 2B). In accord with this, there was no difference in levels of total or phosphorylated p53 (Figure 2E). Thus, the lower tumor burden of *Zfp148*-deficient mice is not caused by reduced growth rate. However, the p53-activity was increased in tumors from old *Zfp148^gt/+^* mice (24-26 weeks old) compared to age-matched controls, raising the possibility that *Zfp148* deficiency reduces progression of advanced tumors (Figure 2F).

**Figure 3:**
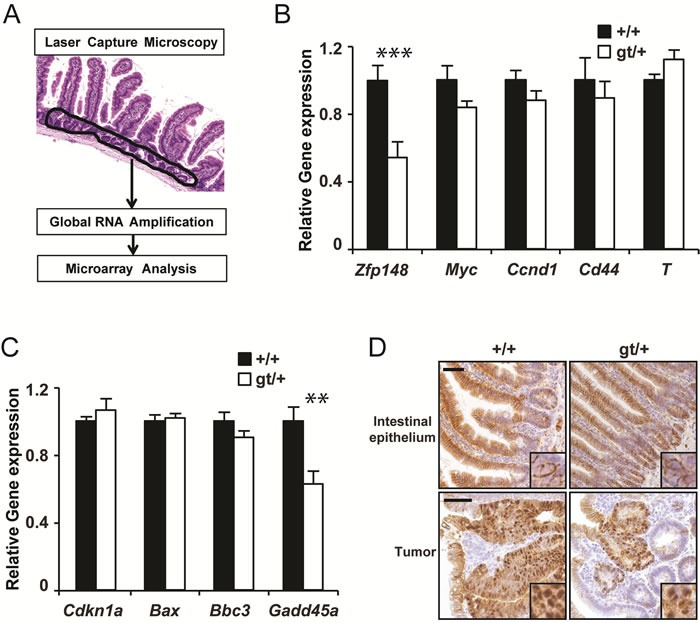
*Zfp148* deficiency does not affect basal expression of p53 or β-catenin target genes in crypt epithelium **A.** Schematic representation of the gene expression analysis procedure. **B.**-**C.** mRNA levels of *Zfp148* and four p53-target genes **B.** and four β-catenin target genes **C.** in laser microdissected crypt epithelium from *Apc*and *ApcZfp148* mice at 12 weeks of age (*n* = 6). **D.** Representative immunohistochemistry micrographs showing β-catenin (brown) in normal intestinal epithelium (top) and in tumors (bottom) in *Apc*and *ApcZfp148* mice at 12 weeks of age. Inserts show subcellular localization of β-catenin. Sections are counter stained with Mayers hematoxylin. Data are represented as mean ± SEM. *P < 0.05, **P < 0.01. Scale bars, 50μm.

An alternative possibility is that *Zfp148* deficiency affects expression of key genes in the crypt epithelium where new tumors are initiated. Tumor initiation is driven by β-catenin and suppressed by p53 [14, 18]. To address this, we used laser capture microscopy and gene expression arrays to extract transcriptional profiles from normal crypts (Figure 3A). However, we could not detect any concordant effects of *Zfp148* deficiency on β-catenin or p53 signaling in normal crypts, as judged by expression analysis of individual β-catenin or p53 target genes or gene set enrichment analysis (GSEA) of known signaling pathways (Figure 3B, 3C and Supplementary Table 1). Moreover, immunohistological analysis did not reveal any difference in the amount or subcellular localization of β-catenin between *Zfp148^gt/+^*intestines and controls (Figure 3D). Thus, *Zfp148*-decifiency does not affect the activation of β-catenin or p53 in normal crypts.

Tumor initiation is triggered by loss of the second *APC* allele which leads to constitutive activation of β-catenin. To test whether Zfp148 plays a role downstream of activated β-catenin, we treated intestinal explants from *Zfp148^gt/+^* mice and *APC^Min/+^* controls with a pharmacological inhibitor of glycogen synthase kinase 3β (GSK3β) for 6 and 20 hours. Phosphorylation of β-catenin by GSK3β is required for proteasomal destruction of β-catenin and inhibition of GSK3β activates β-catenin.

**Figure 4:**
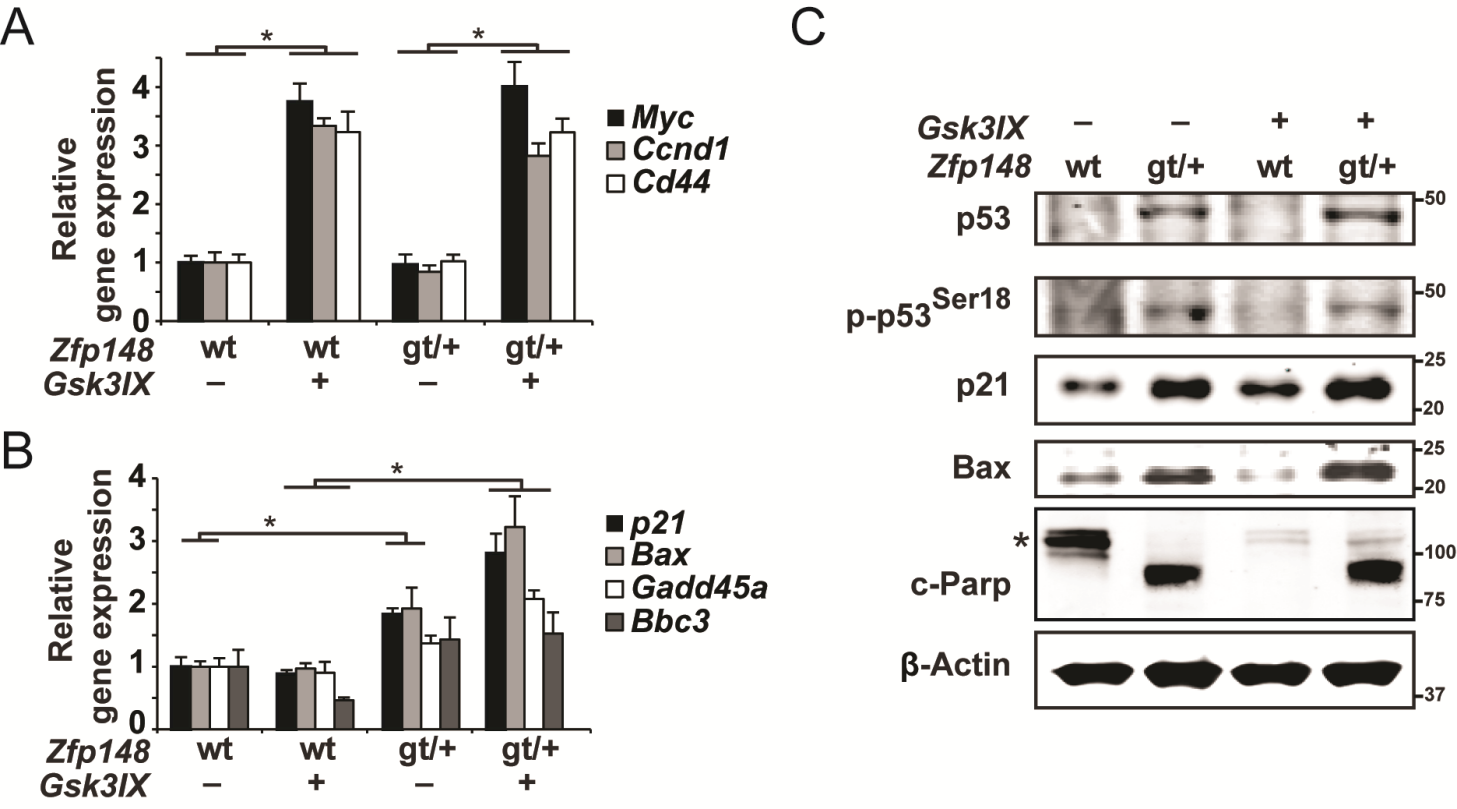
Constitutive activation of β-catenin induces p53-activation and apoptosis in small intestine explants from *ApcZfp148* mice **A.**-**B.** Taqman RT-PCR assessment of mRNA levels of three β-catenin target genes **A.** and four p53-target genes **B.** in small intestine explants that were dissected from *Apc*and *ApcZfp148* mice and treated with 2μM of the GSK3β-inhibitor GSK3IX or DMSO. The explants in **A.** were treated for 6 hours and those in **B.** for 20 hours (*n* = 6). **C.** Western blots of phosphorylated and total p53, the p53 targets p21 and Bax, and the apoptosis marker cleaved Parp1 (89-kD fragment) in small intestine explants that were dissected from *Apc*and *ApcZfp148* mice and treated with 2μM GSK3IX or DMSO for 20 hours. Asterisk indicates uncleaved Parp1 (116-kD fragment). β-Actin was used as loading control. Data are represented as mean ± SEM. *P < 0.05, **P < 0.01.

As demonstrated by the induction of β-catenin target genes, β-catenin was activated in the explants 6 hours after GSK3β inhibition, but did not affect the expression of p53 target genes at this time point (Figure 4A and Supplementary Figure 3A, 3B). However, p53 targets were robustly induced in explants from *Zfp148^gt/+^* mice at 20 hours after GSK3β inhibition (Figure 4B). Importantly, GSK3β inhibition had no effect on p53 target genes in *APC^Min/+^* controls. Since expression of p53 targets was higher in untreated *Zfp148^gt/+^* explants that were cultured for 20 hours compared to controls (Figure 4A), culture-stress likely contributed to p53-activation in *Zfp148^gt/+^* explants.

In accord with this, protein levels of p53 and p53 target genes and the apoptosis marker cleaved Parp1 were markedly higher in explants from *Zfp148^gt/+^* mice compared to controls (Figure 4C). Collectively, the results suggest that *Zfp148* deficiency increases p53 activity in response to constitutively activated β-catenin during tumor initiation, and that newly transformed tumor cells are eliminated by p53-induced apoptosis in *Zfp148*-deficient mice.

**Figure 5:**
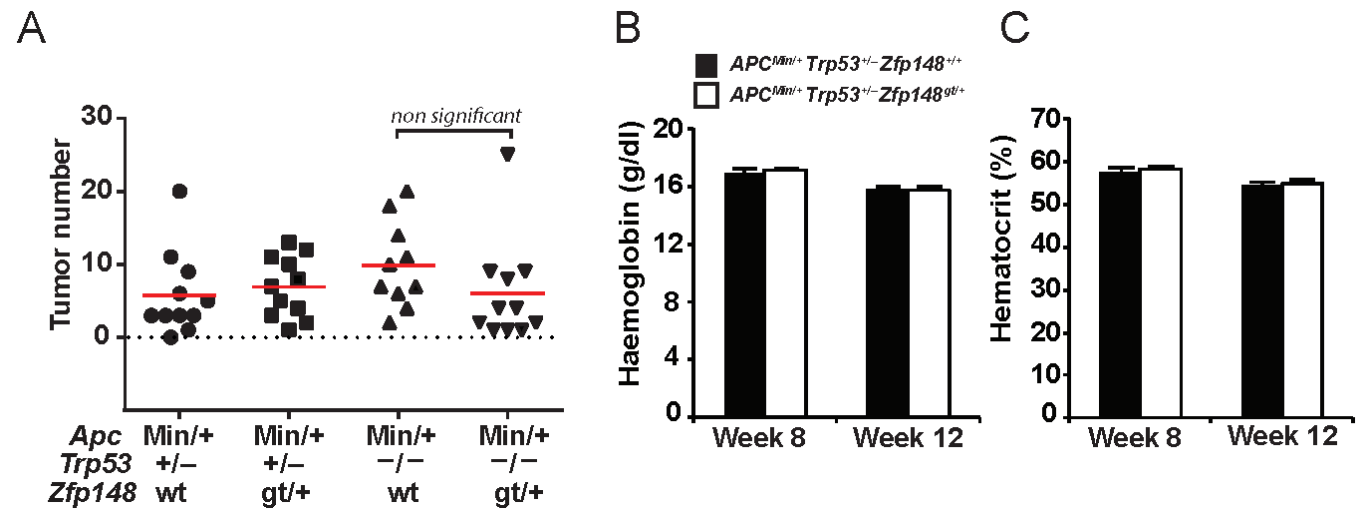
Deletion of one or two copies of Trp53 restores tumor growth in *ApcZfp148* mice **A.** Tumor count in small intestine of *ApcTrp53, ApcZfp148 Trp53, ApcTrp53* and *ApcZfp148 Trp53* mice at 12 weeks of age (*n* = 11). **B.**-**C.** Levels of haemoglobin **B.** and hematocrit **C.** in blood from *ApcTrp53* and *ApcZfp148 Trp53* mice at 8 and 12 weeks of age (*n* = 11). Data are represented as mean ± SEM.

To test this idea, *Zfp148^gt/+^* mice were bred onto *Trp53^+/−^* and *Trp53^−/−^* genetic backgrounds and evaluated for tumor development at 12 weeks of age. There was no difference in tumor count between *Zfp148^gt/+^* and *Apc^Min/+^* controls on *Trp53^+/−^* or *Trp53^−/−^* genetic backgrounds (Figure 5A). Moreover, levels of haemoglobin and hematocrit were indistinguishable between *Zfp148^gt/+^* and *Apc^Min/+^* controls on *Trp53^+/−^* background (Figure 5B, 5C). We conclude that tumor development is restored by deletion of one or both copies of *Trp53*, and that *Zfp148* deficiency suppresses tumor formation by increasing p53 activity. Survival of *Zfp148^gt/+^* mice on p53-deficient background was not investigated since *Trp53^+/−^* and *Trp53^−/−^* mice develop other types of neoplasia that may confound the result [19, 20].

We finally investigated whether p53 is inactivated by somatic mutations in *Zfp148*-deficient tumors. We did not find any mutations in exons 5-8 of the *Trp53* gene in 5+5 tumors from *Zfp148^gt/+^* mice on *Trp53^+/+^* and *Trp53^+/−^* background, respectively. Since exons 5-8 harbour 90 percent of all *p53*-mutations in human cancers [21, 22], we conclude that functional p53 is retained in the majority of *Zfp148*-deficient tumors.

## DISCUSSION

In this study we show for the first time that *Zfp148* deficiency reduces tumor development. Loss of one copy of *Zfp148* markedly reduced tumor frequency in the *APC^Min/+^* model of colorectal carcinoma, extended survival of the mice with 69%, and reduced secondary manifestations of the disease including anaemia and intestinal bleedings. The effect was entirely dependent upon functional p53 showing that *Zfp148* deficiency reduces tumor development by increasing p53 activity.

*Zfp148* deficiency had no effect on cell proliferation or apoptosis in early tumors indicating that Zfp148 does not affect tumor growth rate. Instead, we propose that Zfp148 controls the fate of newly transformed tumor cells by setting a threshold for p53 activation in response to constitutively activated β-catenin. The idea is supported by two lines of evidence. First, levels of phosphorylated and total p53, expression of p53-target genes, and the apoptosis marker cleaved Parp-1 were up-regulated in intestinal explants from *Zfp148^gt/+^* mice in response to pharmacological inhibition of GSK3β. The same treatment had no effect on p53-activity or apoptosis markers in explants from control mice. And second, the effect of *Zfp148* deficiency on tumor development was abolished by deletion of one or both copies of *Trp53*, the gene encoding p53.

Since western blotting revealed increased p53 activity in later-stage adenomas from *Zfp148^gt/+^* mice, *Zfp148* deficiency may also reduce progression of later-stage tumors. Thus, the data suggest a model where Zfp148 promotes tumor initiation, has no function early on in progression, and may be reactivated in later stage tumors.

The rescue of tumor development by deletion of one copy of *Trp53* is in line with previous data. Breeding onto a *Trp53^+/−^* background rescued macrophage proliferation and atherosclerosis in *Zfp148^gt/+^* mice and lung development and neonatal lethality in *Zfp148^gt/gt^* mice [2, 3]. And it restored normal cell proliferation and expression of p53-target genes in *Zfp148^gt/gt^* MEFs [2]. Thus, reducing p53-expression is sufficient to prevent activation of p53 effector pathways in *Zfp148*-deficient mice.

We did not detect any somatic *p53* mutations in tumors from *Zfp148*-deficient mice in spite of strong p53-dependent suppression of tumor development. One possible explanation is that p53-activity is transiently increased in these mice during tumor initiation by loss of APC, and that p53 activity is low at other time points, leaving no time for selection of somatic mutations. In support of this, there was no difference in cell proliferation, apoptosis, or levels of total and phosphorylated p53 in tumors from 12 weeks old *Zfp148^gt/+^* mice compared to controls. Moreover, knockout of p53 has no or little impact on tumor development in *APC^Min/+^* mice on *Zfp148*-wildtype background, suggesting that the selective pressure for accumulating p53 mutations is low in this model [23-25].

The role of Znf148 (human Zfp148 homologue) in human CRC was recently investigated [9]. In familial adenomatous polyposis, expression of Znf148 increased from normal mucosa to adenomas to carcinomas, thus supporting the possibility that Znf148 promotes tumor initiation. Similar results were obtained in sporadic CRC with increased expression in adenomas and carcinomas compared to normal mucosa. Interestingly, the data suggests that Znf148 may play a different role in advanced CRC since Znf148 expression in carcinomas was inversely correlated with TNM stage and patient survival. However, the significance of Zfp148 in advanced tumors remains to be tested in mouse models that develop metastatic CRC.

Cancer chemoprevention is often cited as an effective strategy to reduce cancer mortality. Our finding that global deletion of one copy of *Zfp148* reduces tumor initiation is therefore clinically interesting. Since mice lacking one copy of *Zfp148* appear to be healthy and have a normal lifespan, our finding suggests that therapeutic targeting of Zfp148 could reduce the incidence of CRC by increasing p53 activity without causing detrimental side effects. However, transcription factors such as Zfp148 are not ideal drug targets although significant progress has been made in this field. A more realistic approach may be to target other proteins in the Zfp148-p53 pathway.

The mechanism by which Zfp148 regulates p53 activity is not known [2, 3]. The physical interaction of Zfp148 with p53 raises the intriguing possibility that Zfp148 might regulate p53 directly. However, Zfp148 deficiency up-regulates Cdkn2a in cultured cells opening up for an alternative mechanism [2, 26]. Since deletion of *Wip1* suppresses *APC^Min^*-driven polyposis by increasing p53 activity in response to constitutively activated β-catenin [18], it is possible that Wip1 and Zfp148 operate in the same pathway. Defining the mechanism by which Zfp148 suppresses p53 activation in the intestinal epithelium should be a prioritized area for future studies.

In this study we show that *Zfp148* deficiency reduces tumor formation in the *APC^Min/+^* model. CRC is one of the most prevalent cancer forms in the Western world and a leading cause of cancer-related death. Mutations in the *APC* gene is a major causative event of CRC in humans [12] and identification of modifier genes that affect cancer outcome in the *APC^Min/+^* model is of clinical interest. Because deletion of *Zfp148* reduces the initiation of tumors, preventive targeting of Zfp148 may be efficient to reduce the incidence of CRC and thereby CRC mortality.

## MATERIALS AND METHODS

### Mouse breeding

*Apc^Min/+^* and *Trp53^tm1Tyj^* (Trp53^-^/) mice were obtained from The Jackson Laboratory and *Zfp148^gt/+^* mice were produced by us [2]. The mice were kept on a 129/Bl6 mixed genetic background and all experiments were performed with littermate controls. Genotyping was performed by PCR amplification of genomic DNA from mouse tail biopsies. PCR primers used for genotyping are listed in Supplementary Table 2. Mice were fed on a regular diet and had unlimited supply of food and water. All animal procedures used in this study were approved by The Animal Research Ethics Committee in Gothenburg.

### Histological analyses

Adenoma frequency was compared in 12 week *APC^Min/+^/Zfp148^+/+^*, *APC^Min/+^/Zfp148^gt/+^* and *APC^Min/+^/Zfp148^gt/gt^* mice. Mice were dissected, the intestines were removed and separated into four segments; colon and three segments of the small intestines. The segments were rinsed in PBS and prepared using the Swiss roll technique [27]. Tissues were fixed in 4% formaldehyde, imbedded in paraffin and 5 μm thin sections were stained with Haematoxylin and Eosin. Adenoma counts were performed on one single sagittal section from each segment by investigator blinded to the genotype. Histopathological grade was assessed by a pathologist (O.N.).

### Survival study

Mice were sacrificed when they became moribund, defined as when they became listless, their haematocrit was below 20%, or their bodyweight was reduced by 15%.

### Blood analyses

Blood was drawn from the tail into EDTA-coated tubes (Monovette, Sarstedt). Samples were analysed on a Hemato analyser KX-21N (Sysmex) to determine haemoglobin, hematocrit and leukocyte count.

### Gastrointestinal bleeding

Faecal blood was detected by using Hemoccult test for faecal blood (Beckman-Coulter).

### Immunohistochemistry

Intestinal tissues were fixed in 4% formaldehyde, imbedded in paraffin and sectioned in 5 um thin sections. Staining was performed using standard protocol. Slides were boiled for 10 min in Citric acid (10 mM, pH = 6) to unmask epitopes. Primary antibody anti-PCNA (Santa Cruz, SC-7907) was diluted 1:1000 and secondary antibody (anti-rabbit-Alexa 546, Molecular Probes) was diluted 1:500 and primary antibody anti-β-catenin (BD, 610154), was diluted 1:200 and visualized with Vectastain Elite ABCkit, Peroxidase, Rabbit IgG, (Vector Laboratories) as before [28].

### Cell proliferation and apoptosis

Apoptosis was evaluated with Terminal deoxynucleotidyl transferase dUTP nick end labeling (TUNEL) using the ApopTag^®^ Fluorescence *In Situ* Apoptosis Detection Kit (Millipore). Cell proliferation was evaluated with 5-Bromo-2′-deoxy-uridine (BrdU) Labeling and Detection Kit I (Roche) 2h after intraperitoneal injection of BrdU (75mg/kg) according to manufacturer's description and as before [2, 28].

### Gene expression analysis

Total RNA was extracted from crypt-enriched tissues that were isolated by laser microdissection (PALM) from 10μm thick cryosections of OCT-embedded and snap frozen Swiss roll preparations of small intestines (proximal). RNA concentration was measured with ND-1000 spectrophotometer (NanoDrop Technologies, Wilmington, DE) and RNA quality was evaluated using the Agilent 2100 Bioanalyzer system (Agilent Technologies Inc, Palo Alto, CA). 250 nanograms of total RNA from each sample were used to generate amplified and biotinylated sense-strand cDNA from the entire expressed genome according to the GeneChip^®^ WT PLUS Reagent Kit User Manual (P/N 703174 Rev 1 Affymetrix Inc., Santa Clara, CA). GeneChip^®^ ST Arrays (GeneChip^®^ XXX Gene 2.0 ST Array) were hybridized for 16 hours in a 45°C incubator, rotated at 60 rpm. According to the GeneChip^®^ Expression Wash, Stain and Scan Manual (PN 702731 Rev 3, Affymetrix Inc., Santa Clara, CA) the arrays were then washed and stained using the Fluidics Station 450 and finally scanned using the GeneChip^®^ Scanner 3000 7G. The gene expression data set has been deposited in the GEO repository with accession number GSE77773.

### Microarray data analysis

The raw data was normalized in the free software Expression Console provided by Affymetrix (http://www.affymetrix.com) using the robust (RMA) method first suggested by Li and Wong in 2001 [29, 30]. Subsequent analysis of the gene expression data was carried out in the freely available statistical computing language R (http://www.r-project.org) using packages available from the Bioconductor project (www.bioconductor.org). In order to search for the differentially expressed genes between *APC^Min/+^* and the *Zfp148^gt/+^* groups an empirical Bayes moderated *t*-test was then applied [31], using the ‘limma’ package [32]. To address the problem with multiple testing, the p-values were adjusted using the method of Benjamini and Hochberg [33].

### Gene set enrichment analyses (GSEA)

Differential expression of gene sets was analyzed with the GSEA V2.1.0 software (Broad institute) using gene sets from the KEGG (Kyoto Encyclopedia of Genes and Genomes) repository (c2.cp.kegg.v4.0.symbols.gmt) and default settings.

### Intestinal explant experiments

One 2 cm segment of the distal ileum was collected, as previously described [34]. Intestinal contents from the segment were removed through flushing with cold PBS and the segment was divided into four equal longitudinal parts. Each part was placed in cell culture plates containing growth medium (Dulbecco's modified Eagle's medium with glutamine and pyruvate, 4.5 g/l glucose, 10% fetal calf serum, 100 U/ml penicillin, and 100 μg/ml streptomycin) with or without 2μM GSK3IX inhibitor (Santa Cruz). The plates were then incubated 6-20h at 37°C in 5% CO_2_. The mixture containing the tissue was transferred to Eppendorf tubes and centrifuged at 14,000 rpm for 5 min. The tissue was collected after removal of the supernatant and used for analysis of mRNA or protein expression. All explant experiments were performed on *APC^Min/+^* background.

### Real-time reverse transcription polymerase chain reaction (RT-PCR)

TaqMan assays were performed as described [35] using TaqMan universal polymerase chain reaction mastermix (Life Technologies) and the predesigned TaqMan assays (Life Technologies) listed in Supplementary Table 2.

### Western blot analyses

Protein levels was determined as previously described [36] with antibodies against p53 (SC-6243, 1:500, Santa Cruz), p-p53ser15 (9284S 1:500, Cell Signal), p21 (SC-6246, 1:1000, Santa Cruz), BAX (SC-493, 1:500, Santa Cruz), c-PARP (9542 S 1:500, Cell Signal), and β-actin (2228, 1:20000, Sigma Aldrich).

### Trp53 sequencing

Exons 5-8 of mouse Trp53 were PCR amplified from genomic DNA isolated from dissected (Zfp148^gt/+^*Trp53^+/+^*) or laser microdissected (*Zfp148^gt/+^Trp53^+/^*) tumors and subjected to Sanger sequencing. Each amplicon was sequenced twice and scanned for deviations from the refseq sequence. Sequencing was performed by Beckman Coulter Genomics using primers listed in Supplementary Table 2.

### Statistical analyses

Statistics were performed with non-parametric Mann-Whitney test for tumor count; Chi Square test for tumor distribution and histopathological grade; ANOVA test for haemoglobin; *t*-test for hematocrit, cell proliferation, apoptosis, and differential gene expression; and Fisher's exact test for gastrointestinal bleeding. Survival curves were generated using the Kaplan-Meier estimator and statistical significance was calculated using the log rank test (Mantel-Cox). Investigators were blinded to the genotype and values are presented as mean ± SEM. A p-value of < 0.05 was considered statistically significant.

## SUPPLEMENTARY MATERIAL FIGURES AND TABLES






