# Supplementary material for: Targeting Zfp148 activates p53 and reduces tumor initiation in the gut
Source: Oncotarget. 2016 Jul 28;7(35):56183–92. doi: 10.18632/oncotarget.10899 (PMC5302905; doi:10.18632/oncotarget.10899)
Supplement: Supplementary file 1 [file oncotarget-07-56183-s001.pdf]

Targeting Zfp148 activates p53 and reduces tumor initiation in the gut

Supplementary Material

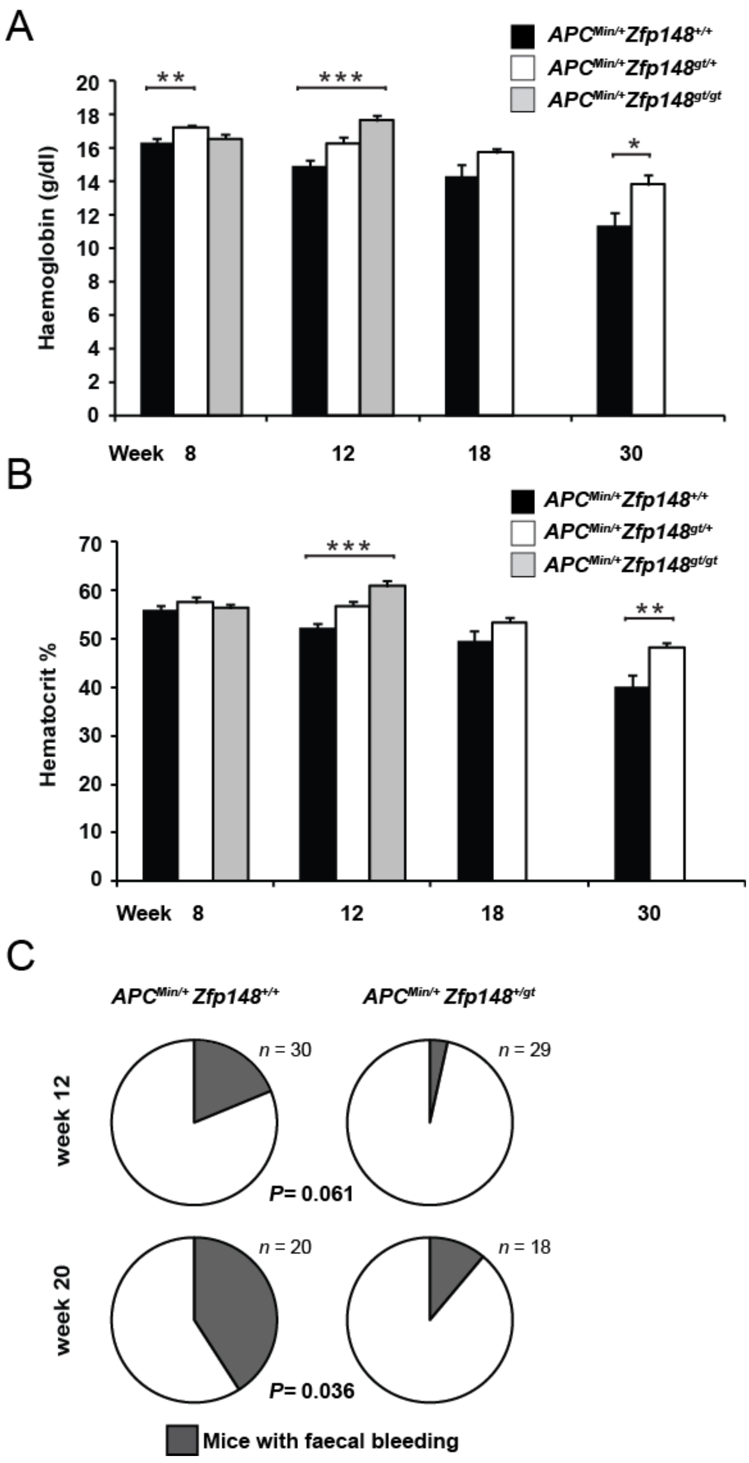

Supplementary Figure 1. (**A–B**) Levels of haemoglobin (A) and hematocrit (B) in blood from *Apc*$^{Min/+}$, *Apc*$^{Min/+}$*Zfp148*$^{gt/+}$, and *Apc*$^{Min/+}$*Zfp148*$^{gt/gt}$ mice at 8, 12, 18 and 30 weeks of

age (n = 12). *Apc*$^{Min/+}$*Zfp148*$^{gt/gt}$ mice were not included at 18 and 30 weeks. (C) Fraction of mice with faecal bleeding at 12 and 20 weeks of age (n = 18-30). Data are represented as mean ± SEM.

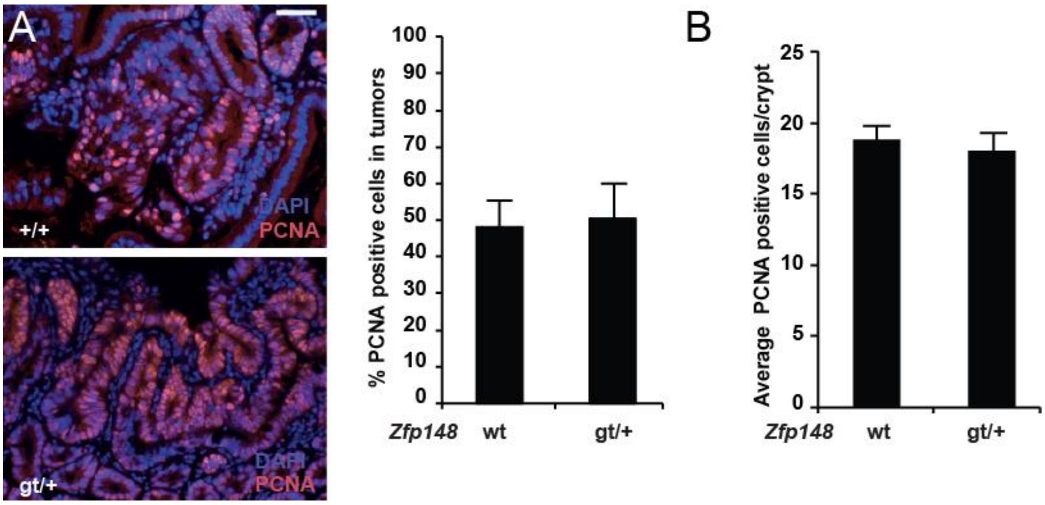

Supplementary Figure 2. (**A**) Left: Representative immunofluorescence micrographs showing PCNA-positive cells in tumor tissue in *Apc*$^{Min/+}$and *Apc*$^{Min/+}$*Zfp148*$^{gt/+}$ mice. Right: Quantification of PCNA-positive cells in tumor tissue (PCNA-positive cells per tumor area). (**B**) Quantification of PCNA-positive cells in normal intestinal epithelium (average number of PCNA-positive cells per crypt). Data are represented as mean ± SEM. Scale bars, 50µm.

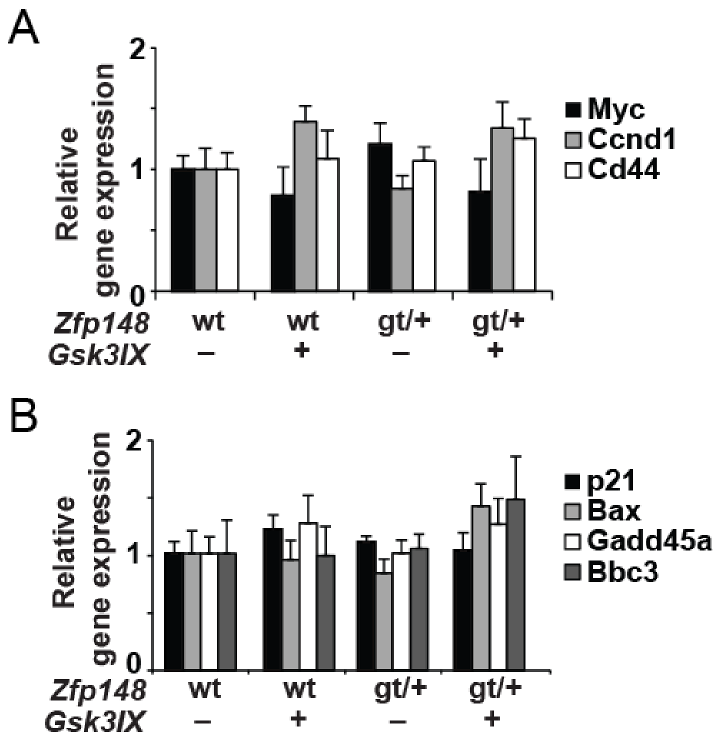

Supplementary Figure 3. (**A-B**) Taqman RT-PCR assessment of mRNA levels of three β-catenin target genes (A) and four p53-target genes (B) in small intestine explants that were dissected from *Apc*$^{Min/+}$and *Apc*$^{Min/+}$*Zfp148*$^{gt/+}$ mice and treated with 2μM of the GSK3β-inhibitor GSK3IX or DMSO. The explants in (A) were treated for 20 hours and those in (B) for 6 hours (n = 6).
